# Supplementary material for: Dynamic corticothalamic modulation of the somatosensory thalamocortical circuit during wakefulness
Source: Nat Commun. 2024 Apr 25;15:3529. doi: 10.1038/s41467-024-47863-8 (PMC11045850; doi:10.1038/s41467-024-47863-8)
Supplement: Supplementary file 1 — Supplementary Information [file 41467_2024_47863_MOESM1_ESM.pdf]

# Supplemental figures to accompany “Dynamic corticothalamic modulation of the somatosensory thalamocortical circuit during wakefulness” By Dimwamwa, E. D. *et al.*

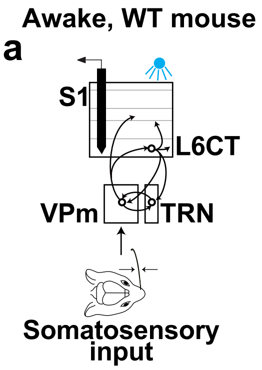


**Figure S1 | The LED input alone does not explain our observed effects of L6CT activation on the cortico-thalamocortical circuit. a.** The direct effect of the LED input alone on ongoing and stimulus-evoked cortical neuron activity is measured from in vivo, silicon probe recordings of neuronal spiking in S1 of an awake, head-fixed, wildtype (WT) mouse with no opsin expression. As in the experiments with opsin-expressing, NTSR1-cre mice, the optical fiber was positioned at the cortical surface and the effect of LED inputs of varying intensities on multi-unit neuron spiking was measured throughout the cortical depth. **b.** Grand PSTHs (mean +/- sem across neurons) of the cortical population in conditions with and without the LED at intensities of 8 (left) and 30 mW/mm² (right). The population's ongoing and sensory evoked activity are unchanged. **c.** The mean change in ongoing firing rate of individual cortical neurons due to LED inputs at 8 mW/mm² (left) and 30 mW/mm² (right), sorted by cortical depth and color-coded according to significance. Significance is determined by the Wilcoxon signed rank test on pre- and post-LED input firing rates across trials. Note that the x-axis range is that shown in Figure S13 for neurons in the NTSR1-cre mice. The spiking of the majority of neurons was unaffected by the LED inputs. **d.** Same as ***c***, but for the sensory response. Note that the x-axis range is that shown in Figure S17 for neurons in the NTSR1-cre mice. The sensory response was unaffected by the LED inputs for all neurons.

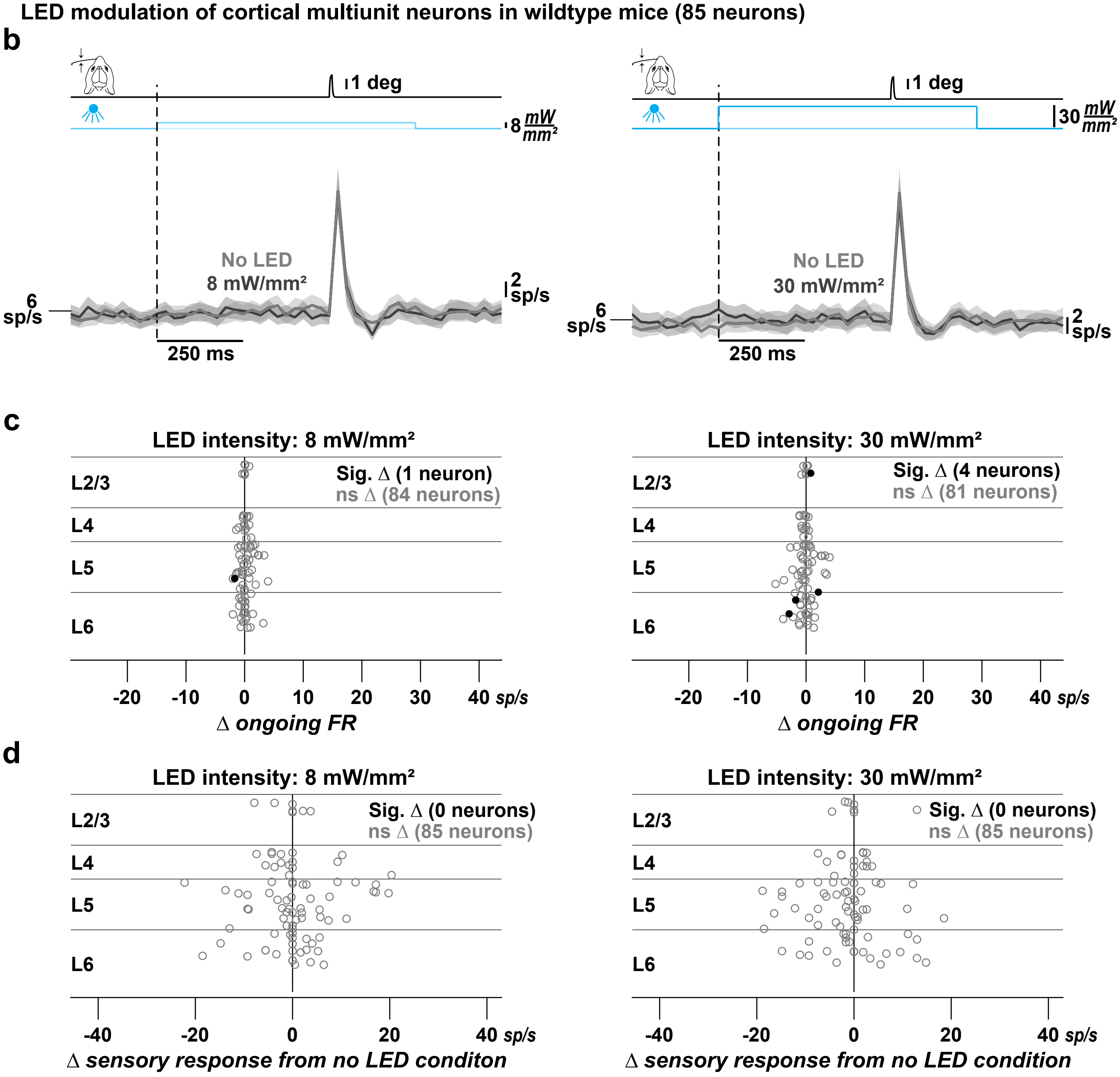

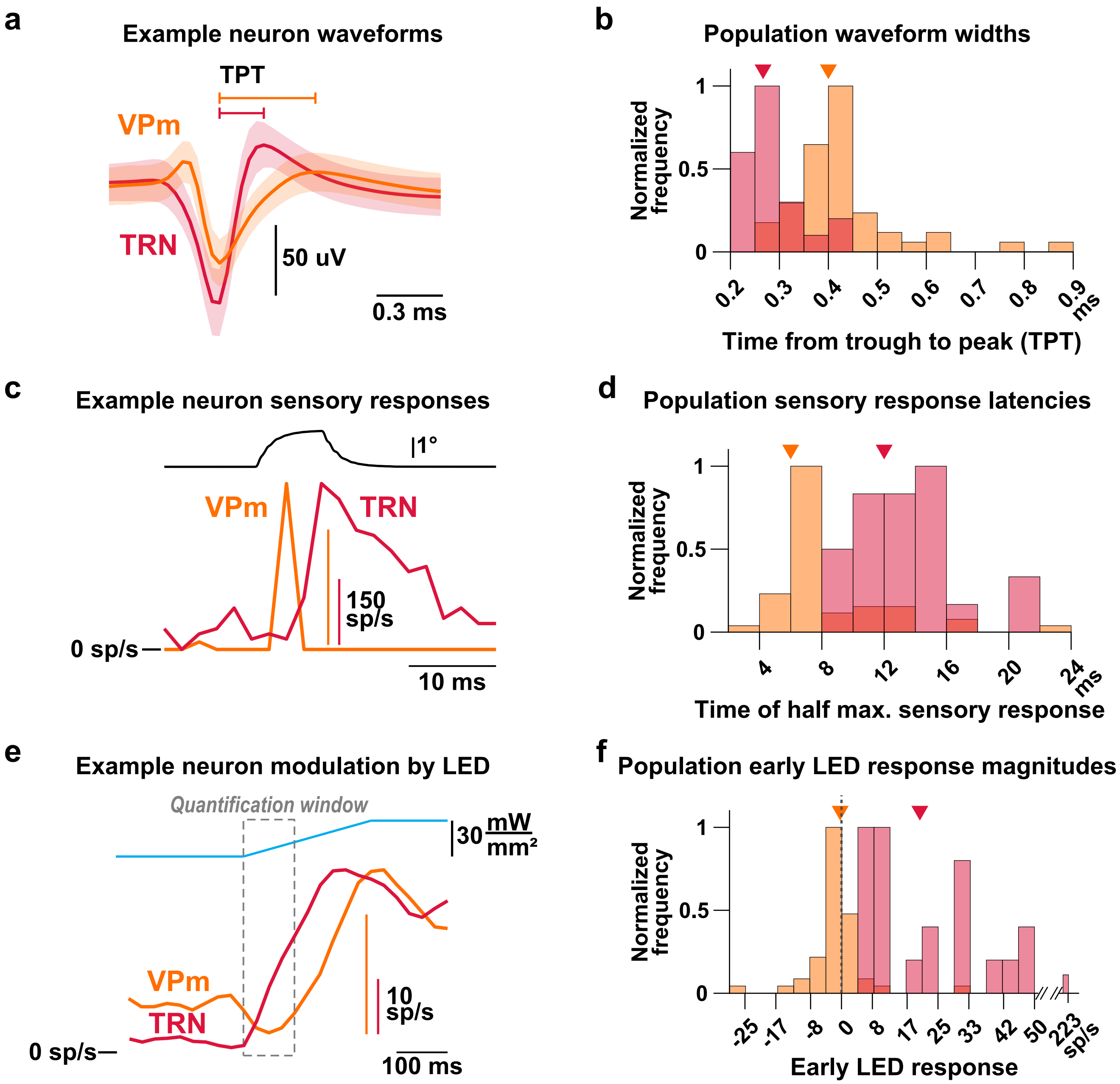


**Figure S2 | Validated using multiple metrics, identified VPm and TRN neurons are sensory-responsive neurons recorded at the relevant stereotaxic coordinate in NTSR1-cre mice. a.** Example extracellular spike waveforms of a VPm and TRN neuron (mean +/- standard deviation). The waveform width, quantified by the trough-to-peak time (TPT; see Materials & Methods), of the example VPm neuron is larger than that of the TRN neuron, as observed by others (Barthó et al., 2014, Guo et al., 2017, Lewis et al. 2015, Makinson et al., 2017). **b.** The normalized distributions of the TPT of all VPm (orange) and TRN (red) neurons have separable peaks (triangle indicates population median), indicating that TRN neurons have more narrow waveform widths. Excluding VPm neurons with TPTs below 0.333 ms and TRN neurons with TPTs above 0.3 ms did not change any reported results. **c.** Sensory response PSTHs of example VPm and TRN neurons. The TRN neuron's sensory response is longer lasting in time compared to that of the VPm neuron, as observed by others (Hartings et al., 2003; Ganmor et al., 2010). **d.** The normalized distributions of the time to half of the maximum sensory response of all VPm and TRN neurons have separable peaks (triangle indicates population median), indicating that TRN neurons have longer response latencies. Excluding VPm neurons with latencies above 8 ms did not change any reported results. **e.** PSTHs of the effect of LED activation of L6CT neurons for example VPm and TRN neurons, both whose spiking is enhanced by 30 mW/mm² optogenetic LED input. However, the enhancement of the spiking in the TRN neuron begins sooner than that of the VPm neuron, which is rather transiently suppressed during the first 100 ms of the LED input. **f.** The normalized distributions of the baseline-subtracted magnitude of the LED modulation in the early window outlined in **e** of all TRN and VPm neurons are well separated (triangle indicates population median). This indicates that the spiking of all TRN neurons is enhanced during the first 100 ms of the LED input, whereas a large majority of VPm neurons are either suppressed or modestly enhanced. Excluding VPm neurons above a cutoff of 4 sp/s did not change any reported results.

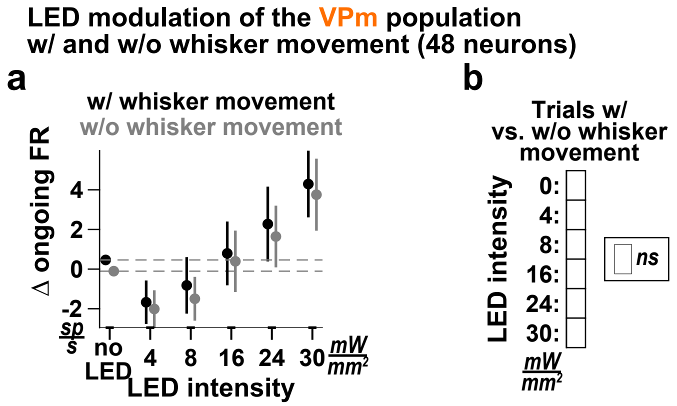


**Figure S3 | The effect of the LED activation of L6CT neurons on VPm spontaneous activity is nearly identical for trials with and without whisker movement. a.** The mean +/- sem change in ongoing activity of the VPm population at the various LED intensities in trials with (black) and without (gray) whisker movement. For this analysis, we only included experiments for which there was a minimum of five trials across all LED intensities for both whisker movement conditions. The gray data points thus reflect a subset of the neurons in Figure 2. Note that a more strict requirement on the minimum allowable trials does not change the nature of the results. The color indicates the p-value using the two-sided Wilcoxon signed rank test with a Bonferroni correction. **b.** Confusion matrix indicating the significance level of the change in ongoing firing rate of the VPm population observed in ***a*** when comparing between trials with and without whisker movement at each LED intensity.

LED modulation of simultaneously recorded VPm neurons

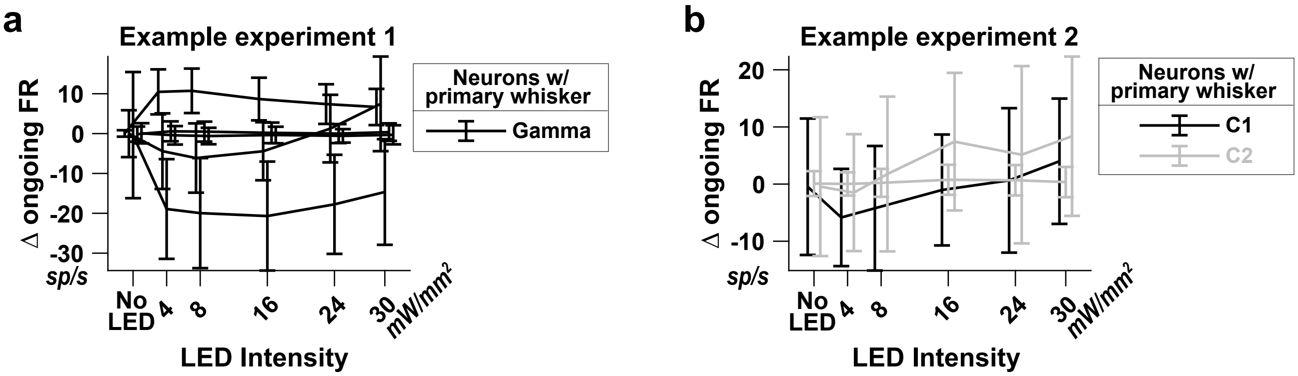


**Figure S4 | The heterogeneity of effects of the LED inputs on the ongoing activity of VPm neurons is observed across the functional topography of VPm. a.** Example mean +/- standard deviation change in ongoing firing rate of simultaneously recorded VPm neurons that have the same primary whisker. Therefore, these neurons receive inputs from L6CT neurons of the same functional cortical column. Yet, the LED modulation effect is variable across these neurons, with one enhanced across all LED intensities, one suppressed, two unmodulated, and one bidirectionally modulated neuron. **b.** Same as a, but for an example recording of VPm neurons from two different primary whiskers, thus receiving inputs from L6CT neurons of differing functional cortical columns. Yet, two of the three neurons are bidirectionally modulated. Importantly, these two neurons have differing primary whiskers.

## LED modulation of individual TRN neurons (27 neurons)

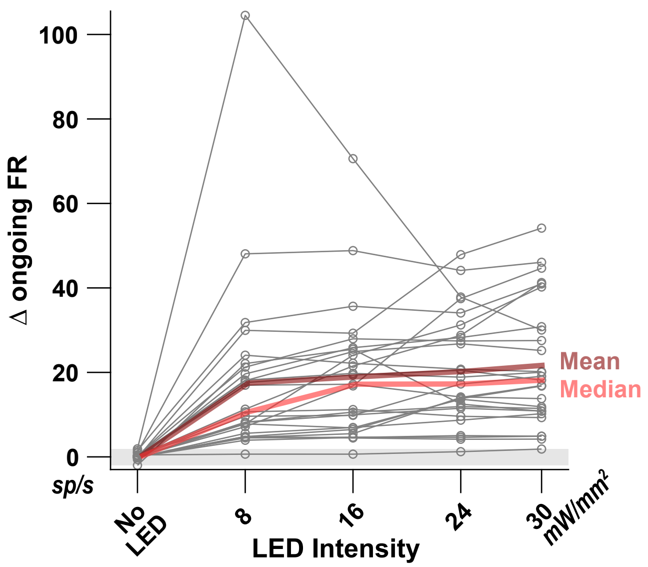


**Figure S5 | The effect of the LED modulation of L6CT neurons on individual TRN neurons.** The mean change in ongoing firing rate of all recorded TRN neurons illustrates relatively homogeneous modulatory effects across the population.

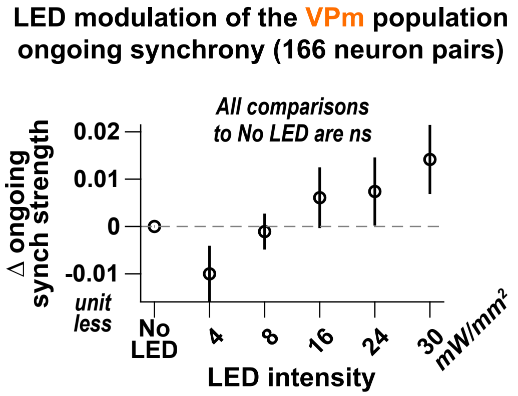


**Figure S6 | The effect of optogenetic activation of L6CT neurons on the synchrony of ongoing VPm activity is small and nonsignificant.** The mean +/- sem change in the VPm population ongoing synchrony strength to ramped LED steps of increasing intensity (statistical comparisons are between the No LED condition and the respective LED-on conditions using the two-sided Wilcoxon signed rank test with a Bonferroni correction).

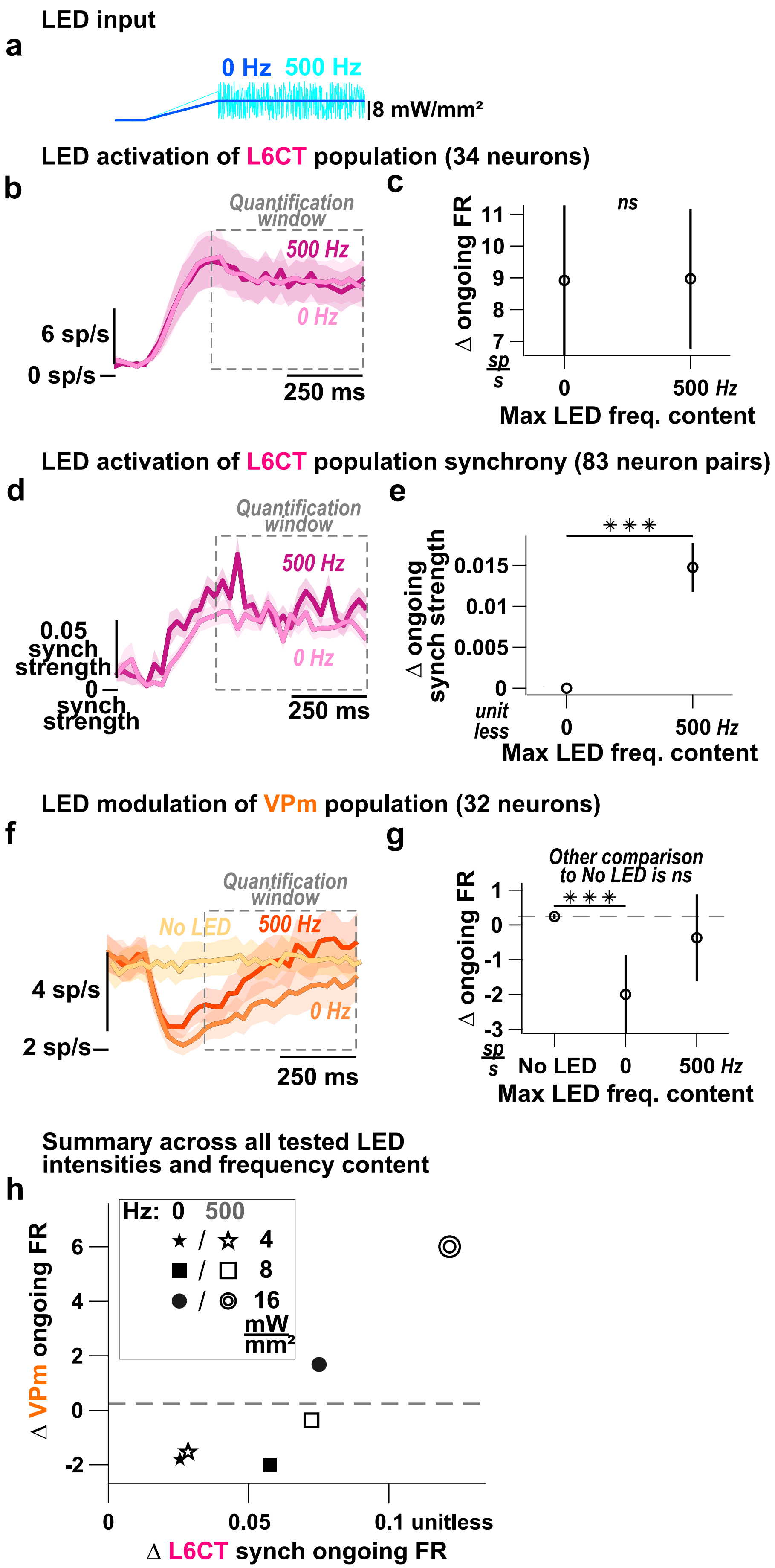


**Figure S7 | Shifts in L6CT population synchrony across firing rates affect the modulatory effect on VPm activity. a.** To further probe the effect of L6CT synchrony on VPm ongoing activity, we presented LED inputs with maximum frequency content of either 0 or 500 Hz, but equal mean of 8 $mW/mm^2$ (instead of 16 $mW/mm^2$ as in Figure 5). Non-frozen realizations of noise were presented across trials. **b.** Grand PSTHs (mean +/- sem across neurons) of the L6CT population activation by the LED inputs in ***a***. Note that the set of neurons analyzed is not the same as those presented in Figures 4 and 5. Also, this dataset has not been parsed to exclude trials where the mouse moved its whiskers. **c.** The mean +/- sem change in ongoing firing rate of the L6CT population contributing to the PSTHs in ***b*** is unchanged between the 0 and 500 Hz LED inputs ($p = 0.12$, determined by the two-sided Wilcoxon signed rank test between the conditions). **d.** Grand PSTHs (mean +/- sem across pairs of neurons) of the L6CT population synchrony strength activation by the LED inputs in ***a***. **e.** The mean +/- sem change in synchrony strength of the L6CT population contributing to the PSTHs in ***d*** increases between the 0 and 500 Hz stimuli ($p = 8.6e{-6}$, determined by the two-sided Wilcoxon signed rank test between the two conditions). **f.** Grand PSTHs (mean +/- sem across neurons) of the VPm population modulation by the LED inputs in ***a***. **g.** The mean +/- sem change in ongoing firing rate of the VPm population contributing to the PSTHs in ***f*** shifts from a significant decrease in response to the 0 Hz stimulus to no change in response to the 500 Hz LED input (0 Hz: $p = 3.8e{-4}$; 500 Hz: $p = 6.7e{-2}$; p-value indicates pairwise comparison to No LED condition using the two-sided Wilcoxon signed rank test with a Bonferroni correction). **h.** Summary of the mean effect of L6CT synchrony across firing rates on the mean change in VPm population firing rate. Same marker shape indicates equal LED intensity (either 4, 8, or 16 $mW/mm^2$). Filled markers indicate the 0 Hz stimulus and hollow markers indicate the 500 Hz stimulus. Increased L6CT population synchrony with equivalent L6CT population firing rate increases the change in the VPm firing rate across multiple L6CT firing rate pairs, although to varying extents across pairs. Taken together, gradual increases in the synchrony strength of the L6CT population shift the modulatory effect on VPm ongoing firing rates from being suppressive to being enhancive.

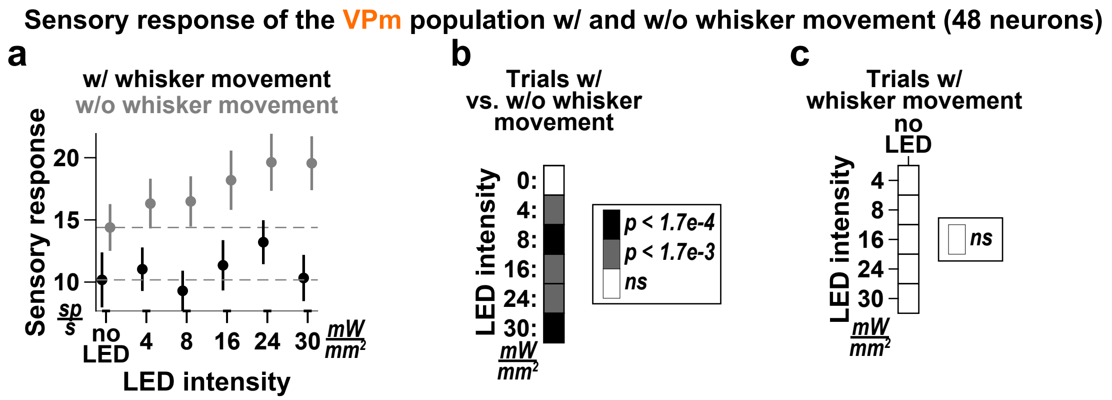


**Figure S8 | The measured effect of L6CT activation on VPm sensory responses in trials with whisker movement is reduced compared to the effect in trials without whisker movement. a.** The mean +/- sem sensory response of the VPm population at the various LED intensities in trials with (black) and without (gray) whisker movement. For this analysis, we only included experiments for which there was a minimum of five trials across all LED intensities for both whisker movement conditions. The gray data points thus reflect a subset of the neurons in Figure 7. Note that a more strict requirement on the minimum allowable trials does not change the nature of the results. **b.** Confusion matrix indicating the significance level of the sensory response of the VPm population observed in ***a*** when comparing between trials with and without whisker movement at each LED intensity. The color indicates the p-value using the two-sided Wilcoxon signed rank test with a Bonferroni correction. **c.** Confusion matrix indicating the significance level of the VPm population sensory response between no LED and LED-on conditions observed only in trials with whisker movement; the color indicates the p-value using the two-sided Wilcoxon signed rank test with a Bonferroni correction. No conditions are different from each other.

**LED modulation of the VPm population sensory response synchrony (166 neuron pairs)**

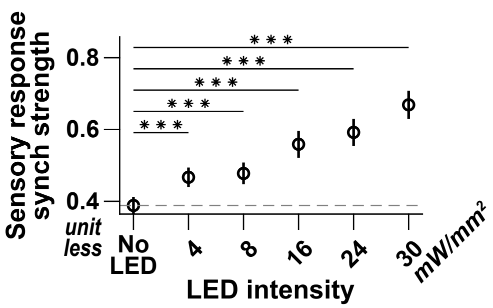


**Figure S9 | Increasing optogenetic activation of L6CT neurons increases the synchrony of VPm sensory responses.** The mean +/- sem VPm population sensory response synchrony strength to ramped LED steps of increasing intensity (statistical comparisons are between the No LED and the respective LED-on conditions using the two-sided Wilcoxon signed rank test with a Bonferroni correction).

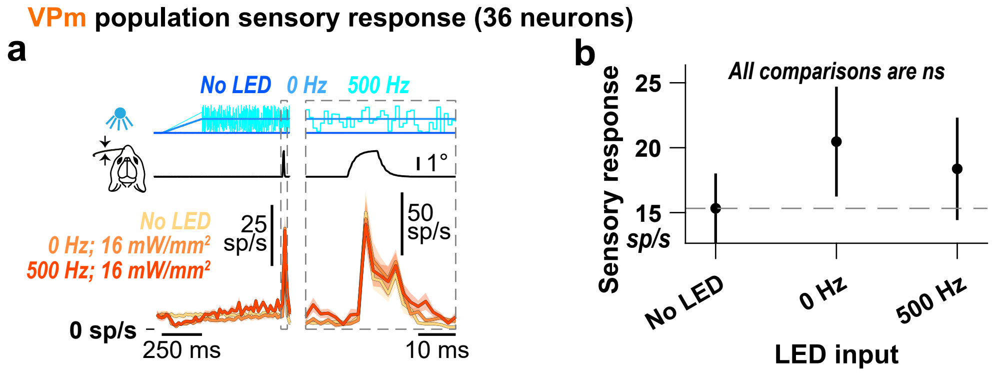


**Figure S10 | Increased L6CT synchrony does not differentially affect VPm sensory responses. a.** Grand PSTHs (mean +/- sem across neurons) of the VPm population sensory response with no LED, the 0 Hz LED step, and the 500 Hz frequency modulated LED input, which selectively increases L6CT synchrony and not firing rate when compared to the 0 Hz LED step. Note that this population is a subset of the population presented in Figure 7. The right panel is an expanded view of the same population's sensory response. Note the change in the vertical scale in the right and left views. **b.** While there is an increasing trend that matches the results presented in Figure 7, the various LED inputs have no significant effect on the baseline-subtracted, stimulus-evoked response of this subset of VPm neurons contributing to the PSTHs in ***a*** (+/- sem). Most relevant, there is no further boost in sensory response between the 0 and 500 Hz conditions (pairwise comparisons are to the No LED or 0 Hz condition using the two-sided Wilcoxon signed rank test with a Bonferroni correction).

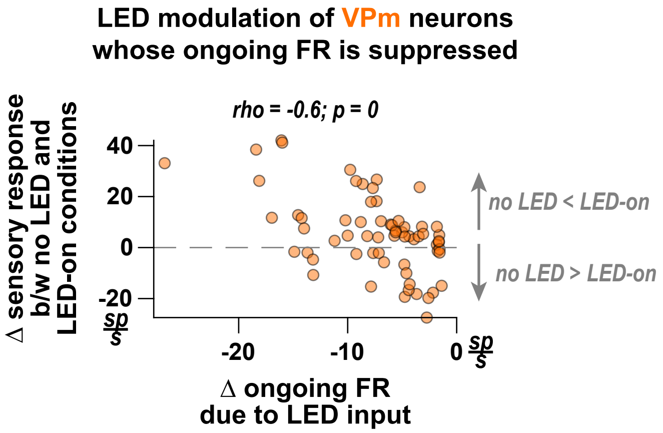


**Figure S11 | The effect of the LED modulation on the ongoing firing rate of VPm neurons does not trivially predict the effect on the neurons' sensory response.** A simple prediction would be that for neurons whose ongoing firing rate is suppressed by the LED inputs, their sensory response would likewise be reduced. Thus, we present the change in ongoing firing rate of individual VPm neurons due to LED activation of L6CT neurons against the difference in sensory response between no LED and LED-on conditions. Note that we only include neurons' activity under conditions where the ongoing firing rate is significantly suppressed by LED inputs using the two-sided Wilcoxon signed rank test. Thus, the set of datapoints represent the response of neurons across all tested LED intensities. Under this set, there is a moderate, negative Spearman's correlation, suggesting a trend where the neurons whose ongoing activity is most suppressed by an LED input rather have a greater sensory response compared to no LED conditions, contrary to the simple prediction. Note that this trend holds even if we only include data under the 4 mW/mm² intensity condition (not shown).

Sensory stimulus-evoked bursting of the VPm population (65 neurons)

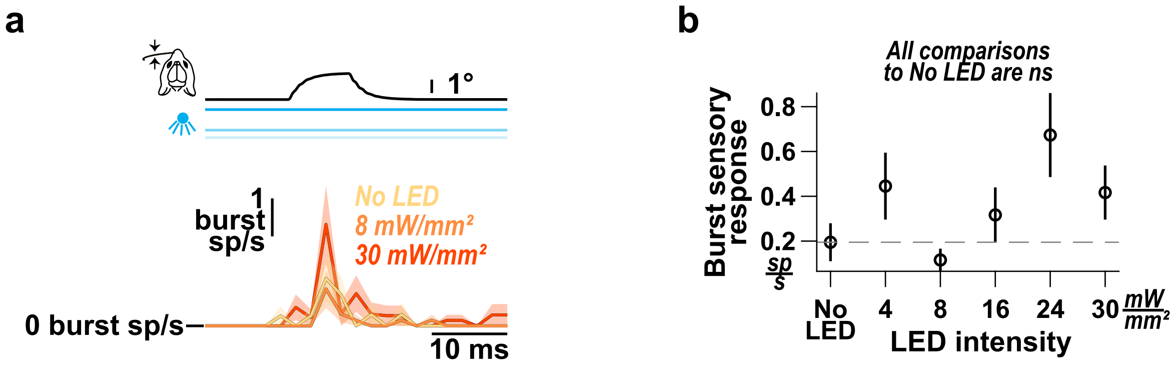


**Figure S12 | The effect of optogenetic activation of L6CT neurons on VPm stimulus-evoked bursting. a.** Grand PSTHs of putative T-Type calcium channel burst spikes (mean +/- sem across neurons) of the VPm neuron population. Bursts are defined as two or more spikes with an inter-spike interval less than 4 ms, preceded by 100 ms or more of no spikes (Borden et al., 2022, Lu 1992, Swadlow & Gusev, 2001, Whitmire & Stanley, 2016, Wright et al., 2021). **b.** Optogenetic activation of L6CT neurons has no significant effect on the mean, baseline-subtracted, stimulus-evoked burst response of the VPm population contributing to the PSTHs in ***a*** (+/- sem; No LED: 0.2 +/- 0.08 sp/s; 8 mW/mm²: 0.11 +/- 0.05 sp/s, p = 0.27; 30 mW/mm²: 0.44 +/- 0.12 sp/s, p = 0.06; p-value indicates pairwise comparison to No LED condition using the two-sided Wilcoxon signed rank test with a Bonferroni correction).

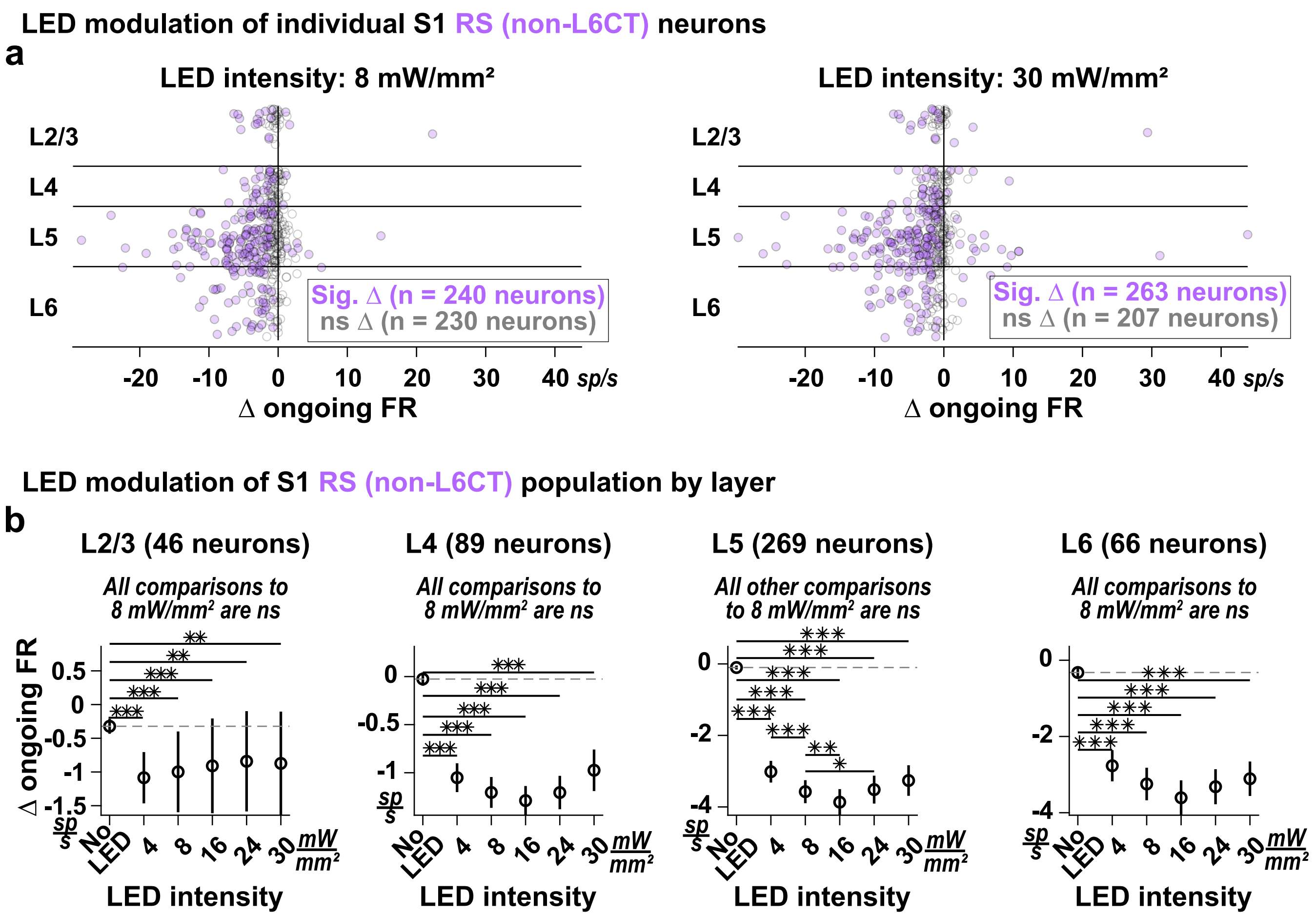


**Figure S13 | Optogenetic activation of L6CT neurons suppresses ongoing activity in S1 RS neurons across all layers. a.** The mean change in ongoing firing rate of individual S1 RS (non-L6CT) neurons due to optogenetic activation of L6CT neurons at 8 mW/mm² (left) and 30 mW/mm² (right), sorted by cortical layer and color-coded according to significance (significance is determined by the Wilcoxon signed rank test on pre- and post-LED input firing rates across trials). Across all cortical layers, we observe neurons whose ongoing activity is suppressed or unchanged by optogenetic activation of L6CT neurons, with few neurons that are enhanced. **b.** Optogenetic activation of L6CT neurons decreases the mean +/- sem change in ongoing firing rate of the RS (non-L6CT) neuron population in each cortical layer. Further, when comparing all other LED conditions to the 8 mW/mm² condition where maximal suppression of VPm ongoing activity was observed, although not significant, a small but apparent reversal in the trend emerges in layers 4, 5, and 6 (p-value indicates pairwise comparison to either No LED condition or 8 mW/mm² condition using the two-sided Wilcoxon signed rank test with a Bonferroni correction; * indicates $p < 5.6e-3$; ** indicates $p < 1.1e-3$; *** indicates $p < 1.1e-4$).

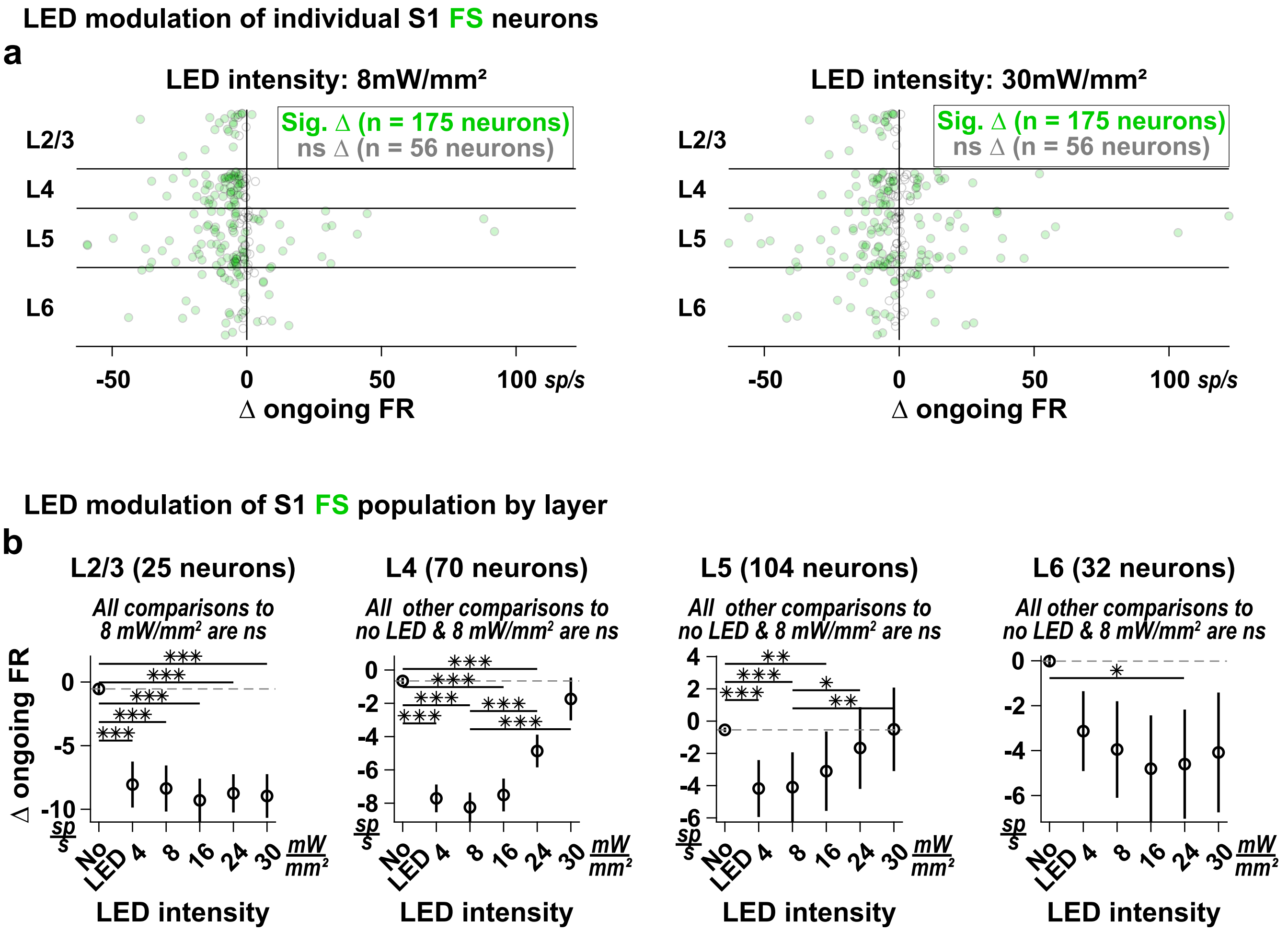


**Figure S14 | Optogenetic activation of L6CT neurons dynamically suppresses ongoing activity in S1 FS neurons in layers 4 and 5. a.** The mean change in ongoing firing rate of individual S1 FS neurons due to optogenetic activation of L6CT neurons at 8 mW/mm² (left) and 30 mW/mm² (right), sorted by cortical layer and color-coded according to significance (significance is determined by the two-sided Wilcoxon signed rank test on pre- and post-LED input firing rates across trials). Across all cortical layers, we observe neurons whose ongoing activity is suppressed or unchanged by optogenetic activation of L6CT neurons. With the exception of layer 2/3, all other cortical layers have neuron's whose ongoing activity is enhanced by optogenetic activation of L6CT neurons, the extent to which depends on the LED intensity. **b.** Optogenetic activation of L6CT neurons decreases the mean +/- sem change in ongoing firing rate of FS populations parsed by cortical layer. Further, when comparing all other LED conditions to the 8 mW/mm² condition where maximal suppression of VPm ongoing activity was observed, a reversal in the trend emerges in layers 4 and 5, such that the effect at 30 mW/mm² is both statistically unchanged from the no LED condition and significantly higher than the effect at 8 mW/mm². Although not significant, a small but apparent reversal also occurs in layer 6 (p-value indicates pairwise comparison to either No LED condition or 8 mW/mm² condition using the two-sided Wilcoxon signed rank test with a Bonferroni correction; * indicates $p < 5.6e-3$; ** indicates $p < 1.1e-3$; *** indicates $p < 1.1e-4$).

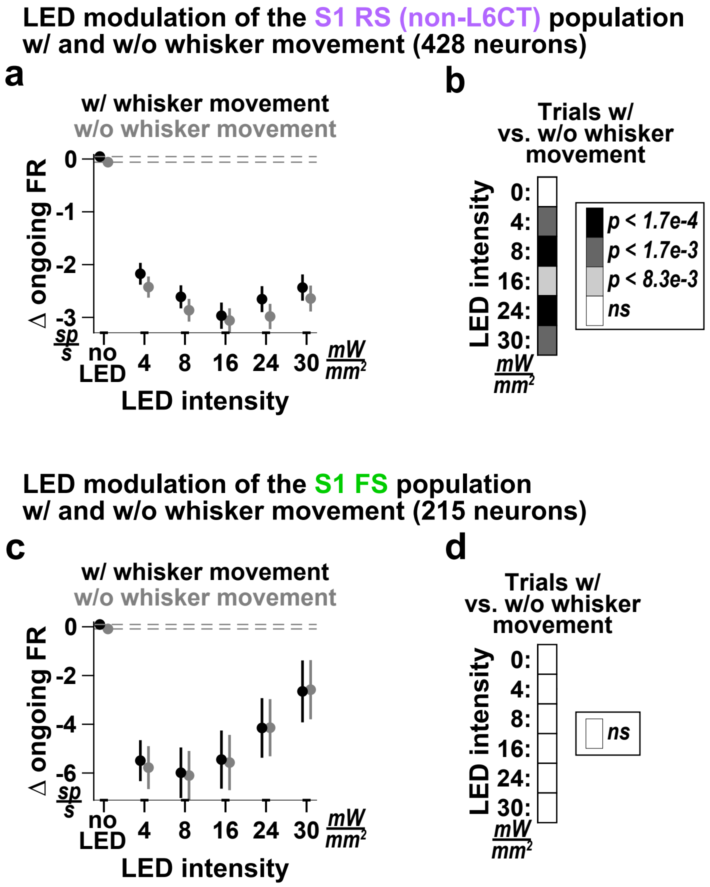


**Figure S15 | Compared to the effect in trials *without* whisker movement, while the measured effect of L6CT activation on ongoing S1 RS activity is modestly decreased in trials *with* whisker movement, the effect on ongoing S1 FS activity is not associated with any change. a.** The mean +/- sem change in ongoing activity of the S1 RS (non-L6CT) population at the various LED intensities in trials with (black) and without (gray) whisker movement. For this analysis, we only included experiments for which there was a minimum of five trials across all LED intensities for both whisker movement conditions. The gray data points thus reflect a subset of the neurons in Figure 8. Note that a more strict requirement on the minimum allowable trials does not change the nature of the results. **b.** Confusion matrix indicating the significance level of the change in ongoing firing rate of the RS population observed in ***a*** when comparing between trials with and without whisker movement at each LED intensity. The color indicates the p-value using the two-sided Wilcoxon signed rank test with a Bonferroni correction. **c-d.** Same as ***a-b***, but for the S1 FS population.

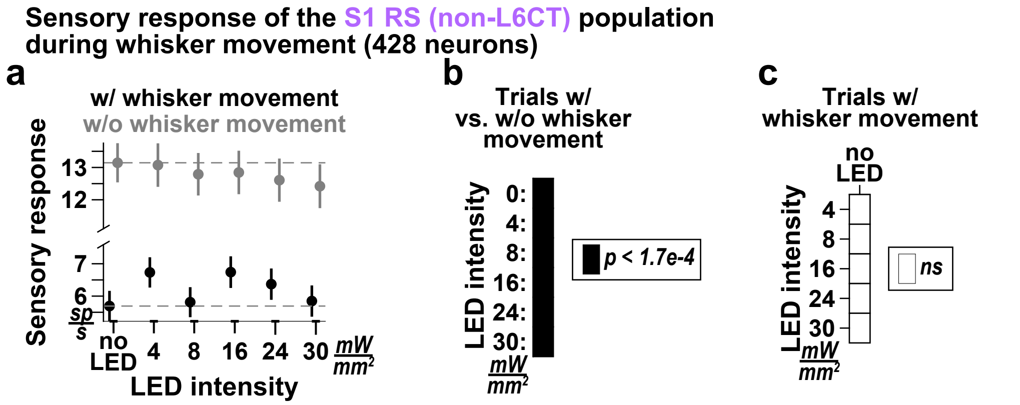


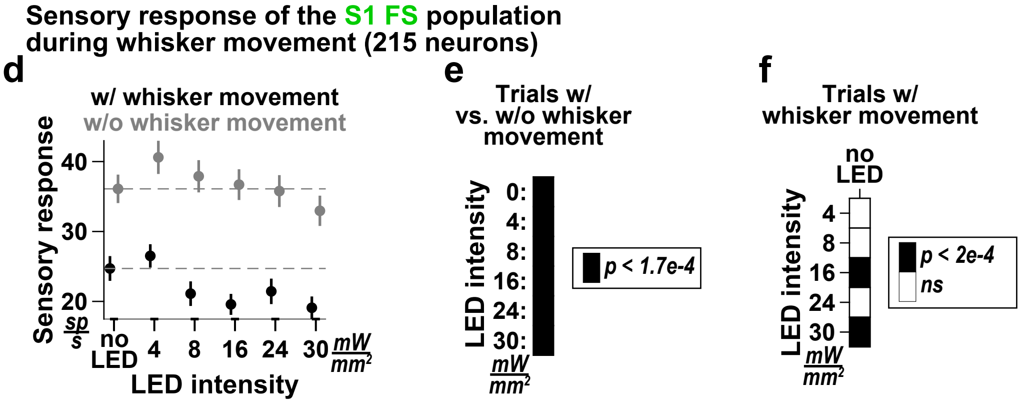


**Figure S16 | The overall impact of L6CT activation on S1 sensory responses is reduced in trials with whisker movement. a.** The mean +/- sem sensory response of the S1 RS (non-L6CT) population at the various LED intensities in trials with (black) and without (gray) whisker movement. For this analysis, we only included experiments for which there was a minimum of five trials across all LED intensities for both whisker movement conditions. The gray data points thus reflect a subset of the neurons in Figure 8. Note that a more strict requirement on the minimum allowable trials does not change the nature of the results. **b.** Confusion matrix indicating the significance level of the sensory response of the RS population observed in ***a*** when comparing between trials with and without whisker movement at each LED intensity. The color indicates the p-value using the two-sided Wilcoxon signed rank test with a Bonferroni correction. **c.** Confusion matrix indicating the significance level of the RS population sensory response between no LED and LED-on conditions observed only in trials with whisker movement; the color indicates the p-value using the two-sided Wilcoxon signed rank test with a Bonferroni correction. No conditions are different from each other. **d-f**. Same as ***a-c***, but for the S1 FS population.

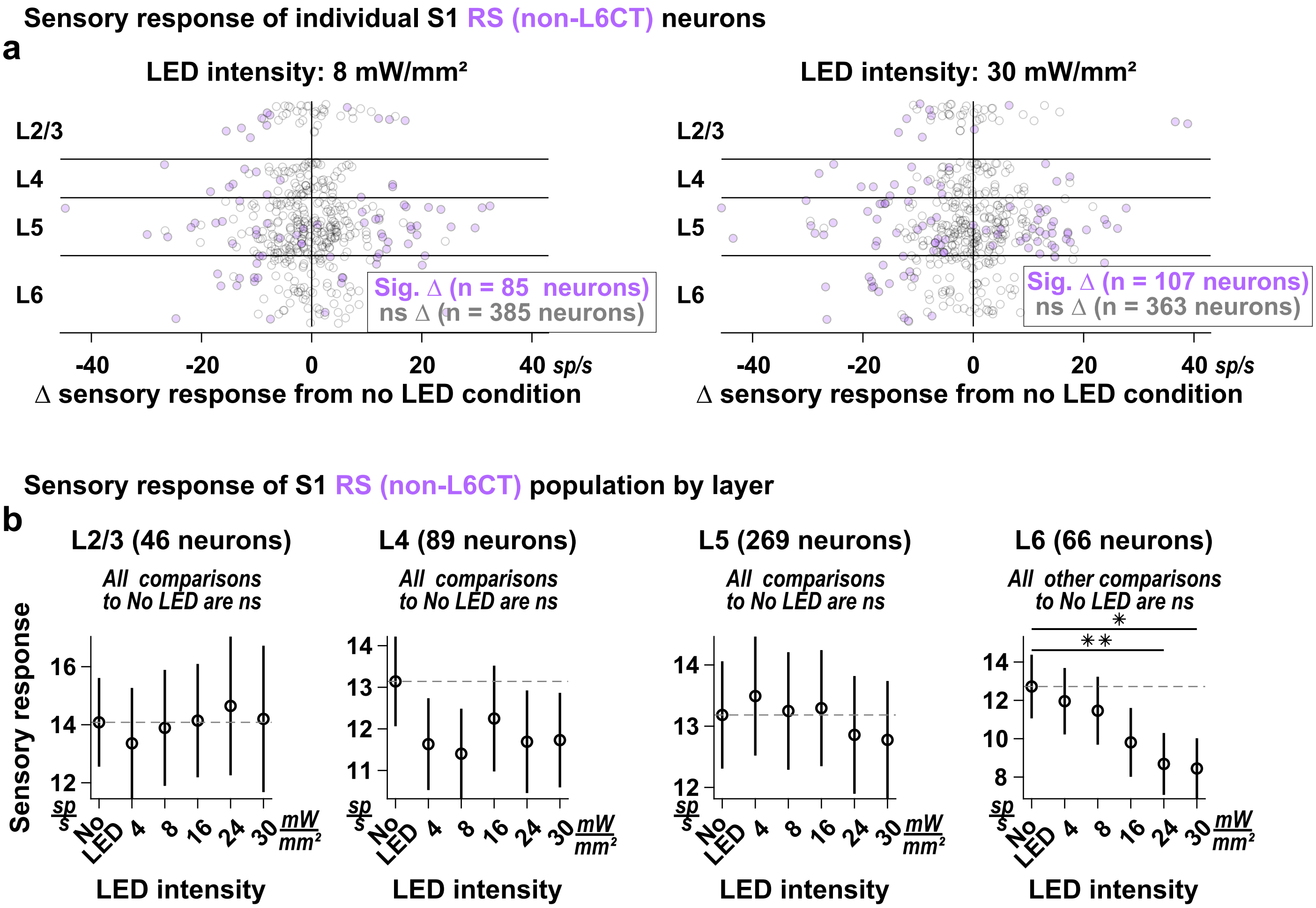


**Figure S17 | Optogenetic activation of L6CT neurons suppresses S1 RS neurons' sensory response in cortical layer 6, leaving the sensory response in all other layers unchanged. a.** The mean change in sensory response from the no LED condition of individual S1 RS (non-L6CT) neurons due to optogenetic activation of L6CT neurons at 8 mW/mm² (left) and 30 mW/mm² (right), sorted by cortical layer and color-coded according to significance (significance is determined by the two-sided Wilcoxon rank sum test on the baseline-subtracted sensory response magnitude across trials in no LED versus LED-on trials). Across all cortical layers, we observe neurons whose sensory response is enhanced, suppressed, and unchanged by optogenetic activation of L6CT neurons. **b.** When parsed by cortical layer, optogenetic activation of L6CT neurons has no effect on the mean +/- sem change in ongoing firing rate of RS (non-L6CT) neuron populations in any layer except layer 6, where sensory responses are suppressed (p-value indicates pairwise comparison to No LED condition using the two-sided Wilcoxon signed rank test with a Bonferroni correction; * indicates $p < 1e-2$; ** indicates $p < 2.0e-3$).

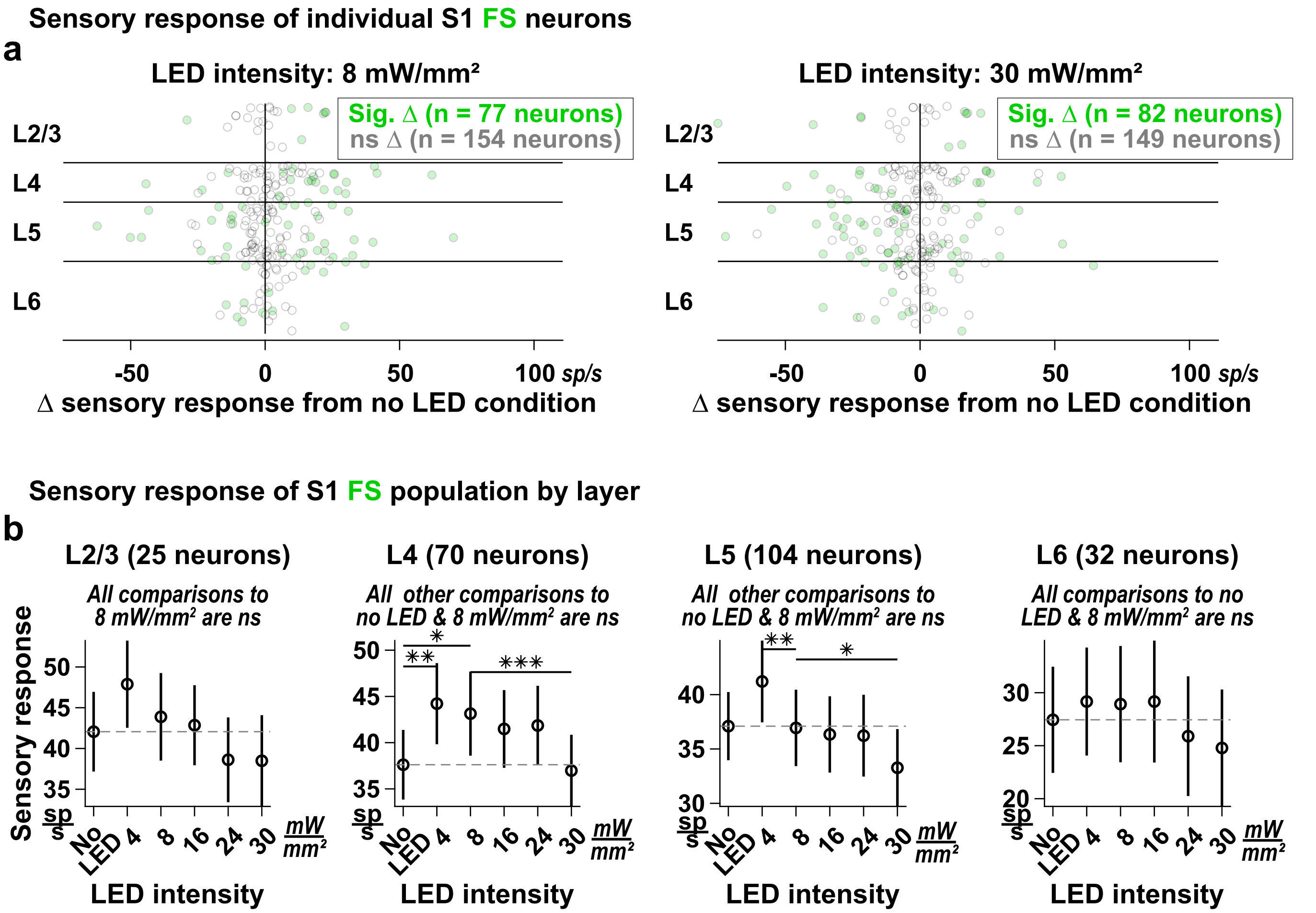


**Figure S18 | Optogenetic activation of L6CT neurons dynamically modulates the sensory response of S1 FS neurons across all layers. a.** The mean change in sensory response from the no LED condition of individual S1 FS neurons due to optogenetic activation of L6CT neurons at 8 mW/mm² (left) and 30 mW/mm² (right), sorted by cortical layer and color-coded according to significance (significance is determined by the two-sided Wilcoxon rank sum test on the baseline-subtracted sensory response magnitude across trials in no LED versus LED-on trials). Across all cortical layers, we observe neurons whose sensory response is suppressed, enhanced, or unchanged by optogenetic activation of L6CT neurons. **b.** Optogenetic activation of L6CT neurons dynamically shapes the mean +/- sem sensory response of FS populations parsed by cortical layer, following the general trend of an enhancement of the sensory response at lower LED intensities (although only significant in layers 4 and 5) and a suppression of the sensory response at higher LED intensities. Although the suppression is not significant in any layer, when comparing all other LED conditions to the 8 mW/mm² condition where maximal suppression of VPm ongoing activity was observed, a reversal in the trend of L6CT activation enhancing sensory responses emerges across all layers (p-value indicates pairwise comparison to either No LED condition or 8 mW/mm² condition using the two-sided Wilcoxon signed rank test with a Bonferroni correction; * indicates $p < 5.6e-3$; ** indicates $p < 1.1e-3$; *** indicates $p < 1.1e-4$).
