# Supplementary material for: Dynamic corticothalamic modulation of the somatosensory thalamocortical circuit during wakefulness
Source: Nat Commun. 2024 Apr 25;15:3529. doi: 10.1038/s41467-024-47863-8 (PMC11045850; doi:10.1038/s41467-024-47863-8)
Supplement: Supplementary file 3 — Reporting Summary [file 41467_2024_47863_MOESM3_ESM.pdf]

nature portfolio

Corresponding author(s): Garrett B. Stanley

Last updated by author(s): March 18, 2024

Reporting Summary

Nature Portfolio wishes to improve the reproducibility of the work that we publish. This form provides structure for consistency and transparency in reporting. For further information on Nature Portfolio policies, see our Editorial Policies and the Editorial Policy Checklist.

Statistics

For all statistical analyses, confirm that the following items are present in the figure legend, table legend, main text, or Methods section.

n/a	Confirmed	
☐	☒	The exact sample size (*n*) for each experimental group/condition, given as a discrete number and unit of measurement
☐	☒	A statement on whether measurements were taken from distinct samples or whether the same sample was measured repeatedly
☐	☒	The statistical test(s) used AND whether they are one- or two-sided<br>*Only common tests should be described solely by name; describe more complex techniques in the Methods section.*
☒	☐	A description of all covariates tested
☐	☒	A description of any assumptions or corrections, such as tests of normality and adjustment for multiple comparisons
☐	☒	A full description of the statistical parameters including central tendency (e.g. means) or other basic estimates (e.g. regression coefficient) AND variation (e.g. standard deviation) or associated estimates of uncertainty (e.g. confidence intervals)
☐	☒	For null hypothesis testing, the test statistic (e.g. F, t, r) with confidence intervals, effect sizes, degrees of freedom and P value noted<br>*Give P values as exact values whenever suitable.*
☒	☐	For Bayesian analysis, information on the choice of priors and Markov chain Monte Carlo settings
☒	☐	For hierarchical and complex designs, identification of the appropriate level for tests and full reporting of outcomes
☐	☒	Estimates of effect sizes (e.g. Cohen's *d*, Pearson's *r*), indicating how they were calculated

Our web collection on statistics for biologists contains articles on many of the points above.

Software and code

Policy information about availability of computer code

Data collection	All digital and analog output signals were controlled by custom software written in MATLAB and Simulink (Version 2015b; MathWorks, Natick, Massachusetts, USA), which is available upon request. Electrophysiology data was collected using either the Cerebus acquisition system (Blackrock Neurotech, Salt Lake City, Utah) or the TDT RZ2 Bioamp Processor (Tucker Davis Technologies, Alachua, FL). Videography was acquired using either of the following CCD cameras: DMK 21BU04.H USB 2.0 monochrome industrial camera (The Imaging Source, LLC, Charlotte, NC) or EoSens CL MC1362 (Mikrotron, Germany). The data generated in this study can be accessed in the Zenodo database: https://doi.org/10.5281/zenodo.8156087. Source data are provided with the manuscript.
Data analysis	As detailed in the manuscript, spike sorting was conducted using KiloSort2 (Pachitariu et al., 2023) and manual curation was conducted using Phy2 (Rossant & Harris, 2013). Whisker motion energy was extracted from acquired videos using FaceMap (Syeda et al., 2022). All other analyses were performed with custom MATLAB programs, which can be accessed in the Zenodo database: https://doi.org/10.5281/zenodo.8156087.

For manuscripts utilizing custom algorithms or software that are central to the research but not yet described in published literature, software must be made available to editors and reviewers. We strongly encourage code deposition in a community repository (e.g. GitHub). See the Nature Portfolio guidelines for submitting code & software for further information.

Data

Policy information about availability of data

All manuscripts must include a data availability statement. This statement should provide the following information, where applicable:

- Accession codes, unique identifiers, or web links for publicly available datasets
- A description of any restrictions on data availability
- For clinical datasets or third party data, please ensure that the statement adheres to our policy

The processed data used to generate all figures in this study have been deposited in the Zenodo database and can be accessed here: https://doi.org/10.5281/zenodo.8156087. Source data are provided with this manuscript. The raw data can be made available upon request.

Research involving human participants, their data, or biological material

Policy information about studies with human participants or human data. See also policy information about sex, gender (identity/presentation), and sexual orientation and race, ethnicity and racism.

Reporting on sex and gender	N/A
Reporting on race, ethnicity, or other socially relevant groupings	N/A
Population characteristics	N/A
Recruitment	N/A
Ethics oversight	N/A

Note that full information on the approval of the study protocol must also be provided in the manuscript.

Field-specific reporting

Please select the one below that is the best fit for your research. If you are not sure, read the appropriate sections before making your selection.

☒ Life sciences ☐ Behavioural & social sciences ☐ Ecological, evolutionary & environmental sciences

For a reference copy of the document with all sections, see nature.com/documents/nr-reporting-summary-flat.pdf

Life sciences study design

All studies must disclose on these points even when the disclosure is negative.

Sample size	Sample sizes were chosen in accordance with the standard number of animals and neurons in comparable studies in the field (e.g. Kirchgessner, M. et al., 2020; Clayton, K. et al., 2021). The exact number of mice and neurons for each analysis is given in the corresponding figure.
Data exclusions	Exclusion criteria are described in detail in the Methods section. In brief, only well-isolated, sensory-responsive neurons were considered for analysis.
Replication	All results represent average activity across many neurons recorded from multiple animals. Specifically, for cortical data, 36 recordings were conducted in 11 mice. For VPm data, 26 recordings were conducted in 13 mice. For TRN recordings, 9 recordings were conducted in 6 mice. As a result, the detailed effects are representative of each neuron population, and not an individual animal or recording.
Randomization	All analyses are performed with a within-subject design, whereby each experimental condition being compared consists of the same pool of neurons. Thus, randomization was not relevant to this study and was not performed.
Blinding	All animals and data were handled in the same way. Stimulus conditions/parameters were presented in a pseudo-random order throughout each recording. Given the within-subject nature of our analyses, no further blinding is relevant to this study.

Reporting for specific materials, systems and methods

We require information from authors about some types of materials, experimental systems and methods used in many studies. Here, indicate whether each material, system or method listed is relevant to your study. If you are not sure if a list item applies to your research, read the appropriate section before selecting a response.

Materials & experimental systems

n/a	Involved in the study
☒	☐ Antibodies
☒	☐ Eukaryotic cell lines
☒	☐ Palaeontology and archaeology
☐	☒ Animals and other organisms
☒	☐ Clinical data
☒	☐ Dual use research of concern
☒	☐ Plants

Methods

n/a	Involved in the study
☒	☐ ChIP-seq
☒	☐ Flow cytometry
☒	☐ MRI-based neuroimaging

Animals and other research organisms

Policy information about studies involving animals; ARRIVE guidelines recommended for reporting animal research, and Sex and Gender in Research

Laboratory animals	Subjects were NTSR1-cre (B6.FVB(Cg)-Tg(Ntsr1-cre)GN220Gsat/Mmcd, MMRRC) or C57BL/J adult mice aged 6 weeks to 6 months.
Wild animals	The study did not involve wild animals.
Reporting on sex	The design of the study consisted of analysis of data from 11 male and 8 female mice. All reported data is combined across sexes.
Field-collected samples	The study did not involve field-collected samples.
Ethics oversight	All experimental and surgical procedures were approved by the Georgia Institute of Technology Institutional Animal Care and Use Committee and were in agreement with guidelines established by the NIH.

Note that full information on the approval of the study protocol must also be provided in the manuscript.
